# Fish waste to sustainable additives: Fish protein hydrolysates alleviate intestinal dysbiosis and muscle atrophy induced by poultry by-product meal in *Lates calcarifer* juvenile

**DOI:** 10.3389/fnut.2023.1145068

**Published:** 2023-03-28

**Authors:** Md Reaz Chaklader, Janet Howieson, Md Javed Foysal, Md Abu Hanif, Hany M.R. Abdel-Latif, Ravi Fotedar

**Affiliations:** ^1^School of Molecular and Life Sciences, Curtin University, Bentley, WA, Australia; ^2^Department of Primary Industries and Regional Development, Fremantle, WA, Australia; ^3^Department of Genetic Engineering and Biotechnology, Shahjalal University of Science and Technology, Sylhet, Bangladesh; ^4^Department of Fisheries Science, Chonnam National University, Yeosu, Republic of Korea; ^5^Department of Poultry and Fish Diseases, Faculty of Veterinary Medicine, Alexandria University, Alexandria, Egypt

**Keywords:** bioactive peptides, animal protein, mucosal barriers, bacteria diversity, muscle atrophy, sustainable aquaculture

## Abstract

Valorising waste from the processing of fishery and aquaculture products into functional additives, and subsequent use in aquafeed as supplements could be a novel approach to promoting sustainability in the aquaculture industry. The present study supplemented 10% of various fish protein hydrolysates (FPHs), obtained from the hydrolysis of kingfish (KH), carp (CH) and tuna (TH) waste, with 90% of poultry by-product meal (PBM) protein to replace fishmeal (FM) completely from the barramundi diet. At the end of the trial, intestinal mucosal barriers damage, quantified by villus area (VA), lamina propria area (LPA), LPA ratio, villus length (VL), villus width (VW), and neutral mucin (NM) in barramundi fed a PBM-based diet was repaired when PBM was supplemented with various FPHs (*p* < 0.05, 0.01, and 0.001). PBM-TH diet further improved these barrier functions in the intestine of fish (*p* < 0.05 and 0.001). Similarly, FPHs supplementation suppressed PBM-induced intestinal inflammation by controlling the expression of inflammatory cytokines (*tnf-α* and *il-10*; *p* < 0.05 and 0.001) and a mucin-relevant production gene (*i-mucin c*; *p* < 0.001). The 16S rRNA data showed that a PBM-based diet resulted in dysbiosis of intestinal bacteria, supported by a lower abundance of microbial diversity (*p* < 0.001) aligned with a prevalence of Photobacterium. PBM-FPHs restored intestine homeostasis by enhancing microbial diversity compared to those fed a PBM diet (*p* < 0.001). PBM-TH improved the diversity (*p* < 0.001) further by elevating the Firmicutes phylum and the *Ruminococcus*, *Faecalibacterium*, and *Bacteroides* genera. Muscle atrophy, evaluated by fiber density, hyperplasia and hypertrophy and associated genes (*igf-1*, *myf5*, and *myog*), occurred in barramundi fed PBM diet but was repaired after supplementation of FPHs with the PBM (*p* < 0.05, 0.01, and 0.001). Similarly, creatine kinase, calcium, phosphorous, and haptoglobin were impacted by PBM-based diet (*p* < 0.05) but were restored in barramundi fed FPHs supplemented diets (*p* < 0.05 and 0.01). Hence, using circular economy principles, functional FPHs could be recovered from the fish waste applied in aquafeed formulations and could prevent PBM-induced intestinal dysbiosis and muscular atrophy.

**GRAPHICAL ABSTRACT:**
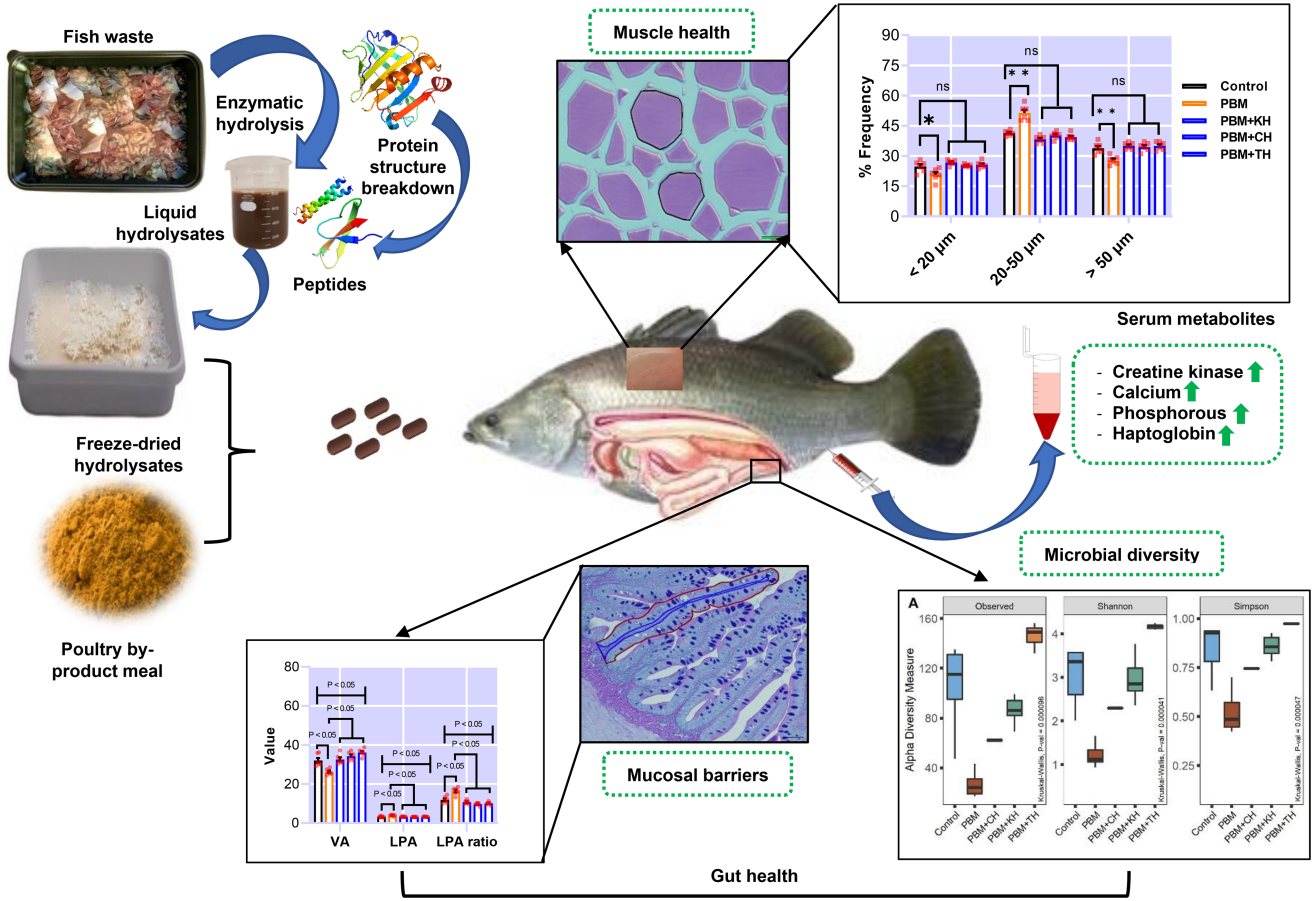

## Introduction

The transformation of fish waste produced by fish processing industries into value-added products such as FPHs, and subsequent utilization in aquafeed, has been an emerging research area in light of the circular economy (1). Such a strategy could potentially result in recycling some of the 1.3 billion tons of food waste produced globally (2) and 7.3 million tonnes waste produced each year in Australia (3). It has been estimated that approximately 100,000 tonnes of waste are produced by seafood industries in Australia every year (4), and an estimated AUD, 15 million *per annum*, is required for disposal (5). High-quality FPHs, characterized by a well-balanced amino acid composition and an abundance of low molecular weight peptides, can be produced from fish processing waste. Such FPH has been considered a suitable immunostimulant for human and animal nutrition (1, 6). Adding a smaller quantity of FPHs has been reported to enhance the palatability of aquafeed, thereby improving the growth and health of aquaculture species (1, 7). The presence, by supplementation, of sufficient bioavailable nutrients in FPHs, has been reported to compensate for nutritional shortcomings of low-quality alternative plants (7) and animal protein aquafeed ingredients (1, 8).

The feasibility of using alternative animal protein aquafeed sources, including PBM, has been investigated for decades in order to facilitate the replacement of FM, a finite and high-cost conventional protein source (9). PBM has been well researched due to its high protein content and amino acid balance similarity close to FM and higher than plant protein sources (10). There has been significant success in the commercial application of PBM and aligned oil in Australia and New Zealand (10, 11). However, the major reason for the historically limited use of PBM on a world scale, as with many animal by-product proteins, is the variation in nutritional composition from batch to batch and among suppliers. This variation has major implications for aquafeed producers due to affecting the biological quality and utilization of the finished feed (9). There is a good number of studies on incorporating PBM in aquafeed formulation with various successes for finfish and crustacean aquaculture (9). One study reported that barramundi fed with high inclusion levels of PBM demonstrated disruption of mucosa barrier functions in the gills and intestine (11). Mucosal barrier functions are important in maintaining cellular and tissue homeostasis (12–14), thereby maintaining intestinal homeostasis, which is directly linked to growth performance and health status of farmed teleost fish (15, 16). Impairment of the fine-tuned intestinal mucosal barrier aggravates intestinal mucosal permeability, contributing to the invasion of pathogenic bacteria and intestinal inflammation of fish (17). Similar to this reported response of barramundi to PBM-based diets, several other studies found that high inclusion of alternative proteins stimulated intestinal dysbiosis by damaging mucosal barriers and stimulating inflammatory responses (18–21).

Such shortcomings of incorporating low-quality alternative proteins into aquafeed could potentially be overcome by complementing with functional additives such as FPHs. For example, supplementation of FPHs ameliorated the quality of PBM, resulting in improved growth performance, immune response, and gut health of barramundi (1, 8). Similarly, 10% of FPH supplemented with 80% of plant protein in the diet of Atlantic salmon, *Salmo salar*, resulted in a significant increase in growth performance and health (7). Supplementation of FPH also benefited the overall immunity of largemouth seabass, *Micropterus salmoides,* increased anti-inflammatory cytokines (*tgf-β* and *il-10*), and reduced pro-inflammatory cytokines (*il-8*), (22). In alternative protein studies, it is important to understand the interaction between intestinal mucosal barrier functions, microbiota, and anti-inflammatory cytokines in maintaining intestinal homeostasis. This area has not been well-researched in barramundi nutritional studies.

Intestinal mucosal barrier damage and inflammation are reported to be linked with intestinal microbial composition. Like many other factors, the balance of bacterial composition can be reshaped or altered by dietary modification (23, 24), which in turn influences the mucosal barrier maturation and development of fish. Several studies have observed a stimulatory effect of alternative aquafeed protein sources on gut dysbiosis of aquaculture fish (25). It is of interest, therefore, to examine the gut microbial composition when functional ingredients, such as FPHs, are added to ameliorate the quality of low-quality alternative protein. This analysis can be completed with deep surveillance by high-throughput 16S rRNA, a new powerful approach of next-generation sequencing technologies independent of culture-based techniques (23, 26). FPHs supplementation could be a good strategy to maintain intestinal homeostasis by reshaping the bacterial composition since FPHs containing essential amino acids are a good natural source of nitrogen required for the growth of gut microbiota (27). Also, FPHs have been reported to proliferate bacterial composition by accelerating the activity of amino peptides and proteases in bacteria (28). One of the most beneficial effects of FPH is the reduction of pathogenic bacteria with a concurrent increase in the growth of beneficial bacteria, as described in the review of Aloo and Oh (29). Our previous study found that supplementing tuna hydrolysate with PBM enhanced the bacteria diversity in the intestine of barramundi (8). In addition, plant protein influenced opportunistic bacteria, in particular, *Aeromonas* bacteria, in the same species, but the abundance of *Aeromonas* decreased when supplemented with FPHs (30).

In addition to the negative effect of replacing FM with PBM or other alternative protein sources on barramundi gut health, a PBM-based diet also caused a histopathological change, myotome necrosis, in the barramundi muscle (11). Previous studies in our laboratory found negative effects of plant protein on muscle microstructure and creatinine kinase production, associated with muscle damage and thereby impacting the muscle health of barramundi (31, 32). However, no single study is dedicated to understanding the potential effect of FM free diet on the interaction between muscle damaging enzymes, muscle micromorphology, and muscle health-relevant genes. Largely overlooked parameters contributing to muscle growth are hyperplasia (recruitment of new muscle fiber) and hypertrophy (growth of existing fiber), the main determinants of fish growth, which have been reported to be regulated by nutrients in juvenile fish (33, 34). For instance, dietary supplementation of threonine, methionine and tryptophan influences the hyperplasia and hypertrophy of various fish species (33, 34).

The comparison of various FPHs derived from marine and freshwater fish waste was the first attempt to evaluate the efficacy of FPHs in preventing the negative effect of completely replacing FM with PBM. Hence, the present study investigated the effect of various FPHs supplementation with PBM on intestinal homeostasis and muscle growth in juvenile barramundi. Intestinal homeostasis was evaluated by intestinal microbiota composition and anti-inflammatory cytokine expression, while muscle growth was determined by the extent of hyperplasia and hypertrophy. To our knowledge, this is the first study on the effect of PBM on the muscle growth of barramundi specifically and the complementary effect of FPH on preventing negative effects on muscle growth induced by PBM.

## Materials and methods

### Ethical statement

All experimental procedures involving fish were conducted at Curtin Aquatic Research Laboratory (CARL), subsequent to review and approval by the Animal Ethics Committee of Curtin University (ARE2018-37) in strict accordance with the guidelines and regulations in Australia for the care and use of animals.

### Experimental diet and fish husbandry

Details of the feed formulation (Table 1) and fish rearing protocol have been described in our previous articles (1, 11). All hydrolysate produced from fish waste was freeze-dried, and the molecular weight distribution is presented in Table 2. The peptide analysis protocol was determined by size exclusion chromatography of proteins, as illustrated in our previously published article (1). Briefly, five test diets were formulated, including a FM-based diet (Control), a PBM-based diet (PBM) and supplementation of 10% yellowtail kingfish, *Seriola lalandi* hydrolysate (KH), carp hydrolysate, *Cyprinus carpio* (CH) and southern bluefin tuna, *Thunnus maccoyii* hydrolysate (TH) respectively with 90% of PBM (designated as PBM-KH, PBM-CH, and PBM-TH). For feed preparation, all the dried ingredients were weighed, and mixed homogenously; thereafter, oil was added and mixed thoroughly. Distilled water was then gradually added to make a stiff dough which was passed through a mincer to produce pellets. Pellets were dried in an oven for 36 h at 60°C and stored in the refrigerator (4 °C) in plastic bags.

**Table 1:** Feed formulation and nutritional composition of experimental diets from our previous studies (1, 11).

	Diets				
*Ingredients* (*g/100 g*)	Control	PBM	PBM + KH	PBM + CH	PBM + TH
^e^PBM^a^	0.00	69.50	61.40	61.40	61.40
^g^KH	0.00	0.00	9.50	0.00	0.00
^h^CH^b^	0.00	0.00	0.00	9.00	0.00
^i^TH^b^	0.00	0.00	0.00	0.00	11.80
^f^Tuna FM^c^	72.00	0.00	0.00	0.00	0.00
Canola oil^c^	1.00	3.00	3.00	3.00	3.00
Cod liver oil^c^	0.50	6.00	6.00	6.00	6.00
Corn/wheat starch^c^	7.00	7.00	3.00	5.00	5.00
Lecithin—Soy (70%)^c^	1.00	1.00	1.00	1.00	1.00
Vitamin C^c^	0.05	0.05	0.05	0.05	0.05
Dicalcium phosphate^c^	0.05	0.05	0.05	0.05	0.05
Methionine	0.00	0.40	0.00	0.00	0.00
Wheat (10 CP)^c^	16.90	11.50	13.50	12.00	9.20
Vitamin premix^c^	0.50	0.50	0.50	0.50	0.50
Cholesterol^c^	0.00	0.00	2.00	2.00	2.00
Salt (NaCl)^c^	1.00	1.00	0.00	0.00	0.00
***Proximate composition* (*dry matter*)**^d^					
Crude Protein (%)	47.88	47.86	48.00	47.71	47.87
Crude Lipid (%)	10.59	12.71	10.73	10.71	10.66
Moisture					
Ash					

^a^Kindly provided by Derby Industries Pty Ltd T/A, Talloman Lot Lakes Rd, Hazelmere WA 6055. ^b^SAMPI, Port Lincoln, Australia. ^c^Specialty Feeds, Glen Forrest Stockfeeders, 3150 Great Eastern Highway, Glen Forrest, Western Australia 6071. ^d^Determined according to Association of Official Analytical Chemists (AOAC). ^e^Poultry by-product meal (PBM): 67.13% crude protein, 13.52% crude lipid and 13.34% ash. ^f^Fishmeal: 64.0% crude protein, 10.76% crude lipid and 19.12% ash. ^g^Kingfish hydrolysate (KH): 57.07% crude protein, 31.98% crude lipid, 2.55% moisture and 0.29% ash. ^h^Carp hydrolysate (CH): 59.01% crude protein, 12.89% crude lipid, 0.73% moisture and 46.21% ash. ^i^Tuna hydrolysate (TH): 58.4% crude protein, 1.05% crude lipid and 11.3% ash.

**Table 2:** Molecular weight (%) of kingfish hydrolysate (KH), carp hydrolysate (CH), and tuna hydrolysate (TH) (1).

Molecular weight (Da)	Hydrolysates		
	KH	CH	TH
>10,000	5.5	3.2	1.8
10,000–5,000	1.9	11.1	6.8
5,000–1,000	22.3	45.0	36.8
1,000–500	31.5	13.1	22.7
500–238	18.1	15.9	16.8
<238.2	20.7	11.6	15.2

Following acclimatization in aligned experimental systems, 25 fishes were stocked in replicated tanks containing 250 L sea water (375 fish in 15 tanks). Water quality, including DO, temperature, and other parameters, was maintained at an optimal level by an aerator, electric heater, and external bio-filter. Ammonia nitrogen and nitrite were checked by commercial kits and maintained at <0.50 mg L^−1^ by water exchange. Experimental diets were fed to fish until visual satiety twice daily, at 8.00 am and 6.00 pm, respectively, over a period of 42 days. After 42 days post-feeding, fish were bulk-weighed and counted to estimate the growth performance of fish.

### Histological analysis

Two hindgut and muscle tissue samples per tank were collected from euthanised (AQUI-S®, 175 mg/L) fish that were used for other biometry indices reported in our previous trials (1, 11). Tissue samples were immediately fixed in 10% neutral buffered formalin and slides were prepared following standard protocol for histological analysis. Hindgut tissue was stained with Periodic Acid-Schiff (PAS) to visualize neutral mucus-producing mucin cells, while muscle tissues were stained with Hematoxylin and Eosin (H&E). All slides were subjected to light microscopy (BX40F4, Olympus, Tokyo, Japan) to take images. Hindgut mucosal morphology were determined by ImageJ software.

### Gene expression analysis

The collected intestinal and muscle tissue from the euthanized (175 mg/L AQUI-S®) barramundi were kept in Eppendorf containing RNA-later (Thermo Scientific, United States) and immediately stored at −80°C until analysis. All genes expression analysis associated with intestinal inflammation (*tnf-α* and *il-10*), intestinal defense (*i-mucin c*), and muscle growth (*igf-1, myf5*, and *myog*) were conducted in the present study. The forward and reverse primers were designed from the known nucleotide sequence of barramundi, *Lates calcarifer*. During primer design from the predicted sequence, the primer sequences were checked and confirmed using the sequence read achieve (SRA) blast in NCBI (National Center for Biotechnology Information). All the primer sequences with their product size, melting temperature (Tm), and GenBank accession number were given in Table 1. The expression analysis was performed by Quantitative real-time PCR following the method described in our previous study (1). Briefly, the protocols of manufacturer’s different test kits including RNeasy Mini Kit (Qiagen, Hilden, Germany), Turbo DNase-free Kit (Thermo Fisher, United States), RNeasy MiniElute Cleanup Kit (Qiagen, Hilden, Germany), and Omnicript RT kit (Qiagen, Hilden, Germany) were followed to extract RNA, digest DNA, purify RNA, and synthesize first strand cDNA, respectively. Following qRT-PCR analysis using PowerUp™ Cyber Green Master Mix (Thermo Scientific, United States) with 7500 Real-Time PCR System (Applied Biosystems, United States), 2^-ΔΔCT^ was used to calculate the relative expression of genes against the *ef-1a* which was more stable than other reference genes (*β-actin*) analyzed in the present study (Table 3).

**Table 3:** Primers of tumor necrosis factor-α (*tnf-α*), interleukin-10 (*il-10*), i-mucin C, insulin like growth factor-1 (*igf-1*), *myf5*, *myog* and elongation factor-1a (*ef-1a*) genes used in qPCR.

Genes	Primer sequence	Tm (°C)	Product size (bp)	Accession no.
*tnf-α*	F: GAGTTTACCACCGGGAATCG	60.5	163	XM_018704368
	R: CCTTTGTCGAACCATCCAGC			
*il-10*	F: CTGATGCCTCACATGGAGTC	60.5	175	XM_018686737
	R: GCAGATCCAGTTCACCCATG			
*i-mucin c*	F: CCAACAACTACTACTGCTGC	58	147	XM_018670824
	R: GGTTGTAAGTGCTGCCATTG			
*igf-1*	F: ACGCTGCAGTTTGTATGTGG	58.4	157	EU136176.1
	R: CTTAGTCTTGGGAGGTGCA	57.5		
*myf5*	F: GCAATGCCATCCAGTACATC	58.4	167	XM_018661930
	R: TGCATTCACCTGTTGCCACA			
*myog*	F: AGGAAGACAGTGACCATGGA	58.4	175	XM_018685841
	R: GCAGCCTTTCGATATACTGG			
*ef-1a*	F: AGGAAGTGAGCACCTACATC	58.4	122	GU188685
	R: CTTGAACCAGGGCATCTTGT			

### Hindgut microbiota

#### Gut DNA extraction

Bacterial DNA from fish samples was extracted using a Blood and Tissue Kit (Qiagen, Hilden, Germany). The DNA extraction was performed following the manufacturer’s instructions with additional beads disruption and homogenization using a tissue lyser II (Qiagen, Hilden, Germany). Briefly, the hindgut digesta were collected inside a biosafety cabinet, transferred into 1.5 mL Eppendorf, and resuspended in 100 μL of physiological phosphate-buffered saline (PBS). After homogenization, samples in Eppendorf were centrifuged briefly, and the protocol of the Blood and Tissue Kit was followed. The DNA concentration was measured in a Nanodrop Spectrophotometer 2000 cc (Thermo Fisher Scientific, United States). An even concentration of 50 ng/μL was achieved through dilution with DEPC-treated water (Thermo Fisher Scientific, Waltham, United States).

#### Amplicon sequencing

The V3V4 region of the 16S rRNA gene was amplified with Illumina overhang adapter in 35 cycles of PCR using a S1000 Thermal Cycler (Bio-Lab Inc., United States) as described previously. Positive amplicons in 1% agarose gel were purified using AMPure Beads, indexed with Nextera® XT Index Kit (Illumina) as described in Illumina 16S Metagenomic Sequencing Library Preparation. Samples were sequenced in a MiSeq platform (Illumina) using V3 kit (600 cycles).

#### Sequence data processing

Raw sequences were imported in qiime2 (v2021.11) for paired-end processing of reads. Deionizing was performed in the DADA2 algorithm implemented in qiime2. Quality trimming of demultiplexed reads was performed using a q2-DADA2 plug-in with the following parameters: -p-trim-left-l 0; -p-trunc-len-f 280; -p-trim-left-r 0; and -p-trunc-len-r 220. Chimeric sequences and reads with more than five expected errors were filtered out. Most non-chimeric reads (92%) were merged and assigned to feature frequency amplicon sequence variants (ASVs) representing the biological feature of amplicon sequences. The feature ASV table was filtered based on the lowest non-zero frequency, 150. Taxonomic classification of ASVs was performed using the consensus BLAST classifier method against SILVA 138 release. Chloroplast and mitochondrial sequences were removed. *Clostridium sensu stricto* 1 was renamed as *Clostridium*, whereas uncultured, unclassified and ambiguous taxa were grouped into other bacteria. Each sample was rarefied to an even depth of 12,376 for further analysis of alpha-beta diversity and microbial composition.

#### Bioinformatic and statistical analysis

Downstream bioinformatic and statistical analysis were performed in R software (v4.22; R Core Team, 2013). Alpha-beta diversity analysis was performed using phyloseq (35), microbiomeSeq,^1^ microbiome,^2^ and vegan R packages. Alpha diversity was measured in terms of observed species, Shannon, and Simpson indices. The Kruskal-Wallis rank test was used to compare diversity measurements among groups. Weighted (relative abundance) and unweighted (presence-absence) UniFrac distance metrics were considered for the visualization of beta-ordination. Permutational multivariate analysis (PERMANOVA) for beta-dispersion with 1,000 permutations was performed using the vegan R package (36) to visualize the feeding effect on gut microbiota. Shared, unique, and core genera in different groups were identified using MicEco, Euler, and microbiome R packages. The relative abundance of bacteria at various taxa levels was calculated with the phyloseq R package. Linear Discriminant Analysis Effect Sizes (LEfSe) at a LDA cut-off value of ≥2.0 was employed to identify differentially abundant bacteria in five different diet groups using the Microbiome Marker R package. *p value* of >0.05 was considered as statistically significant at every phase of the data analysis. The raw sequence data in fastq format have been deposited to the National Center for Biotechnology Information (NCBI) and are currently available under the BioProject accession number PRJNA909982.

### Serum metabolites

Blood collection protocols from three fish/tank were described in our earlier study (1). Following blood collection using from anesthetized barramundi (8 mg/L of AQUI-S®) by puncturing the caudal vein, blood was allowed to clot for 4 h on ice and then centrifuged to separate the serum. The collected sera were preserved at −80°C for further analysis. Creatine kinase (CK; OSR6179) calcium (OSR60117), Mg (OSR6189), inorganic phos (OSR6122), iron (OSR6186), gamma-glutamyltransferase (GGT; OSR6020), and albumin (catalog code OSR6102) were run on a AU480 Clinical Chemistry Analyzer (Beckman Coulter Australia Pty Ltd., Lane Cove West, NSW) using Beckman Coulter clinical chemistry kits. A phase haptoglobin assay kit was used to analyze serum haptoglobin following the manufacturer’s instructions (Tridelta Development Ltd., Co. Wicklow, Ireland).

### Statistical analysis

Unless stated otherwise, all results were represented as mean ± SE. Normality and equal variances of all data were checked by Shapiro-Wilks and Levene’s tests and transformed if necessary. The influence of experimental diets with respect to control were compared by one-way variance analysis (ANOVA) with Dunnett’s multiple comparisons test. The significance level was set at *p* < 0.05, *p* < 0.01, and *p* < 0.001.

## Results

### Intestinal mucosal barriers and inflammatory response

**Figure 1:**
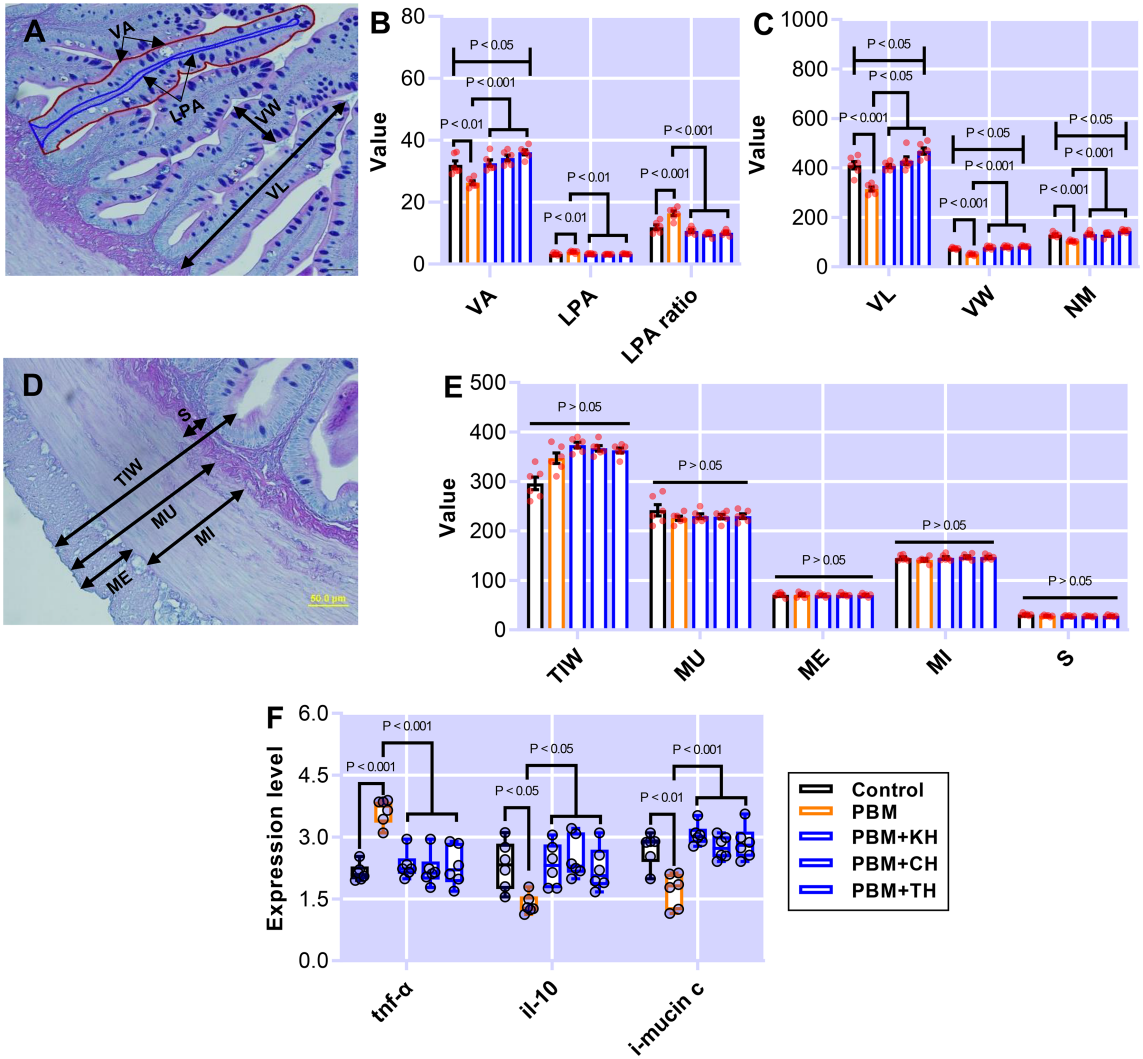
The representative histological micrograph of the intestine showing measurements **(A,D)**; the comparison of quantitative measurements of the intestine **(B,C,E)** and fold change in the expression of pro- and anti-inflammatory cytokines and mucin production relevant gene **(F)** of barramundi fed FM-based control, PBM alone, or supplemented with various FPHs supplemented diets over a period of 42 days. VA, villus area; LPA, lamina propria area; VL, villus length; VW, villus width; NM, neutral mucin; TIW, total intestinal wall; MU, muscularis thickness; ME, muscle interna; MI, muscle ineterna; S, submucosa, *tnf-α*, tumor necrosis factor; *il-10*, interleukin 10, and *i-mucin c*, integument mucin c.

Intestinal quantitative measurements and inflammatory responses in barramundi-fed the various test diets are presented in Figure 1. Barramundi fed a PBM-based diet showed a significantly lower villus area (VA; *p* < 0.01) when compared to those fed the control diet (Figure 1B). Supplementing various FPHs attenuated the negative effects of replacing FM completely with PBM, as supported by comparable VA in barramundi-fed PBM supplemented with FPHs to control fed barramundi (*p* > 0.05) and a significantly higher VA in barramundi-fed PBM-TH (*p* < 0.05) when compared to the PBM-only based diet (Figure 1B). However, lamina propria area (LPA; *p* < 0.01) and LPA ratio (*p* < 0.001) increased significantly in barramundi-fed a PBM-based diet but were similar in barramundi-fed PBM-FPHs based diets when compared with those fed the control diet (Figure 1B; *p* > 0.05). Meanwhile, LPA (*p* < 0.01) and LPA ratio (*p* < 0.001) were significantly lower in barramundi-fed PBM supplemented with FPHs than those fed a PBM-based diet.

Poultry by-product meal-based diet negatively affected the villus length (VL), villus width (VW), and neutral mucin (NM; *p* < 0.001; Figure 1C), which all improved in the intestine of barramundi-fed PBM supplemented with various FPHs when compared with the PBM-based diet (*p* < 0.05 and 0.001). Total intestinal wall (TIW), muscularis thickness (MU), muscularis externa (ME), muscularis interna (MI), and submucosa (S; Figure 1E) were unaffected by test diets (*p* > 0.05).

The expression level of *tnf-α* increased significantly in the intestine of barramundi fed PBM-based diet (*p* < 0.001) but supplementation of FPHs regulated the expression level of *tnf-α*, supported by a comparable expression level of *tnf-α* as observed in control fed barramundi (*p* < 0.001). *il-10*, an anti-inflammatory cytokine, showed a significantly lower expression in the intestine of barramundi fed the PBM-based diet (*p* < 0.05), but the expression levels of *il-10* improved significantly in barramundi fed PBM diets supplemented with FPHs when compared to those fed the PBM-based diet (*p* < 0.05). Barramundi fed PBM supplemented FPHs diets showed a similar expression level of *il-10* when compared with the control (*p* > 0.05). *i-mucin c* showed a similar response to *il-10*, with a lower expression in barramundi-fed the PBM-based diet (*p* < 0.01). *i-mucin c* expression improved when FPHs were supplemented with PBM when compared to those fed only a PBM-based diet (*p* < 0.001). There was no statistical difference in the expression level of *i-mucin c* between the control and FPHs-supplemented PBM groups (*p* > 0.05).

### Gut microbiome

#### Illumina reads

**Figure 2:**
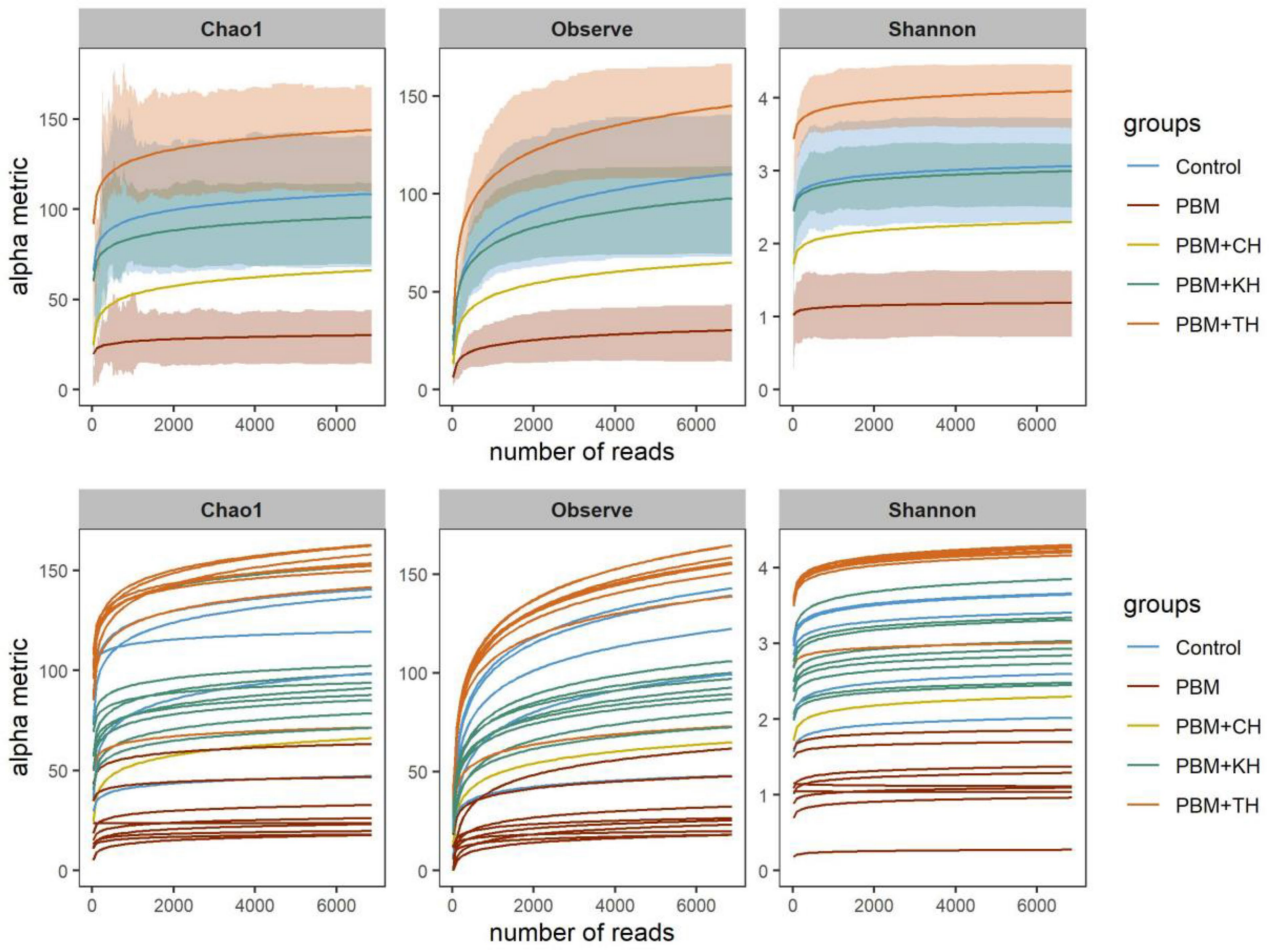
Rarefaction curve showing the depth and saturation level of sequences for a specific group (top) and individual (bottom) sample. Rarefied data was used to generate the rarefaction curve where X-axis represents the number of reads (rarefied) and Y-axis is the values for specific alpha-diversity metrics.

After trimming and filtering, 865,019 quality reads were obtained from 45 samples ranging from 7,864 to 29,862. On average, 19659.2 ± 5047.2 reads were merged, accounting for 86.5% of the total reads generated per sample. In total, 1,215 ASVs were generated, wherein 10 and 20 ASVs comprised 72.6 and 86.2% of the total reads. Phylogenetic classification of reads was obtained for both bacteria and archaea, resulting in 26 phyla, 121 families and 146 genera. The rarefaction curve using rarefied read abundance (Figure 2) and average good’s coverage index (0.998) value suggested sequencing samples at maximum saturation level.

#### Alpha-beta diversity

**Figure 3:**
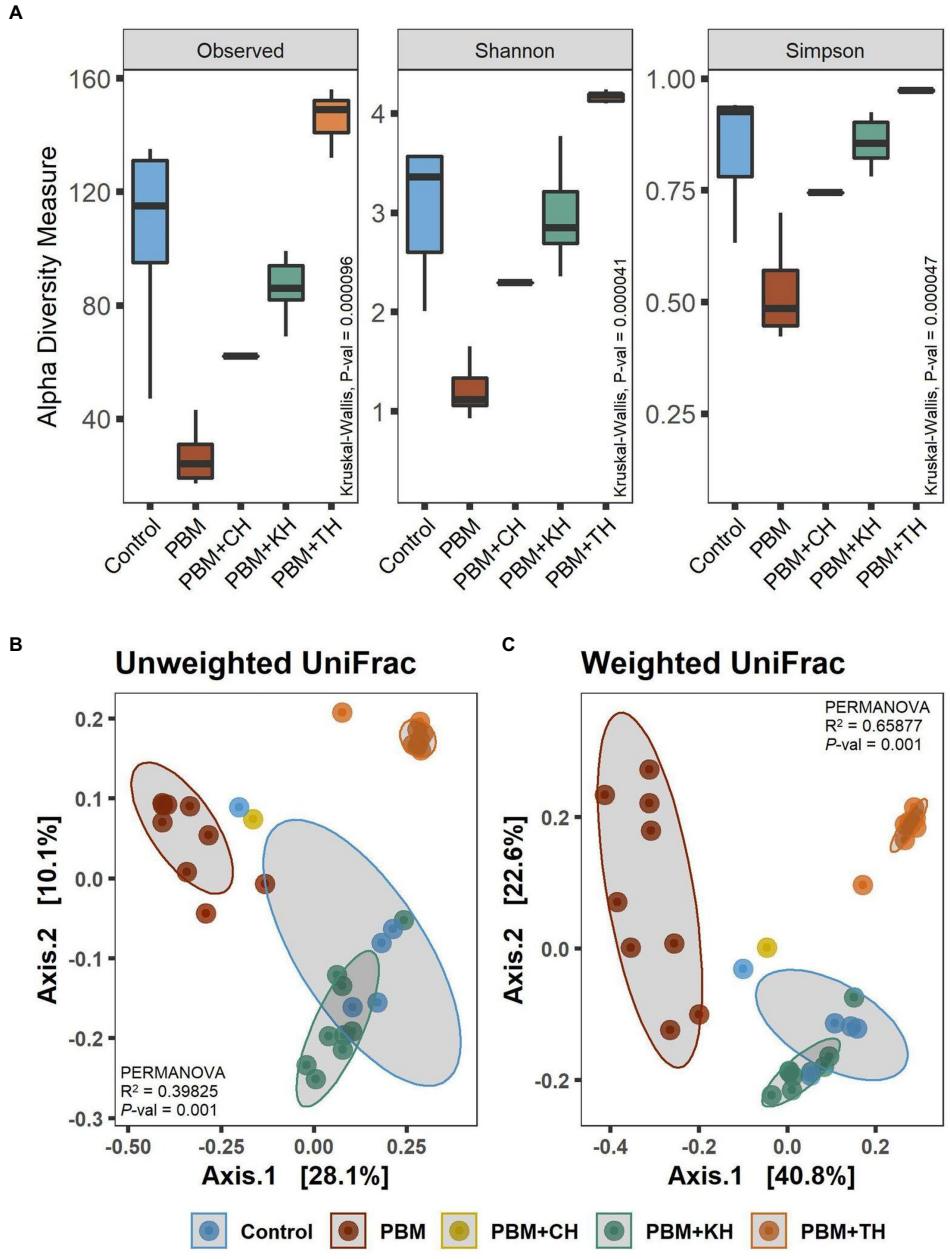
Diversity of gut bacteria in barramundi fed FM-based control, PBM alone or supplemented with various FPHs supplemented diet over a period of 42 days. **(A)** Alpha diversity measurements in terms of Observed, Shannon, and Simpson. **(B)** Beta-ordination presenting clustering of samples based on unweighted (presence-absence) and weighted (relative abundance) UniFrac distance metric. Kruskal-Wallis with Wilcoxon-rank test for multiple comparisons were used to compare alpha-diversity within groups. Different superscript letters in the boxplot indicate significantly different mean values at value of *p* < 0.05.

A comparison of alpha diversity showed significant differences in microbial communities (Figure 3A). The highest bacterial diversity was observed in the fish gut from the PBM + TH diet, whereas the lowest diversity was recorded for PBM only when compared to the other groups (*p* < 0.001). Shannon and Simpson’s analysis revealed significantly higher abundance and even distribution of top abundant taxa in the gut of fish fed the PBM + TH diet (*p* < 0.001). Beta-ordination showed different bacterial communities in the PBM and PBM + TH groups and clustered separately from the control, PBM + KH and PBM + CH diets (*p* = 0.001) regardless of presence-absence (Figure 3B) and relative abundance (Figure 3C). However, relative abundance generated more distinct separation of gut bacterial communities than presence-absence, signifying a diet-specific increase of top abundant existing bacteria in the gut. Centroid analysis of beta-dispersion showed strong influence of PBM and PBM + TH diets in gut bacterial communities’ groups, when compared to the control and PBM + KH which had no distance differences.

#### Microbial composition

**Figure 4:**
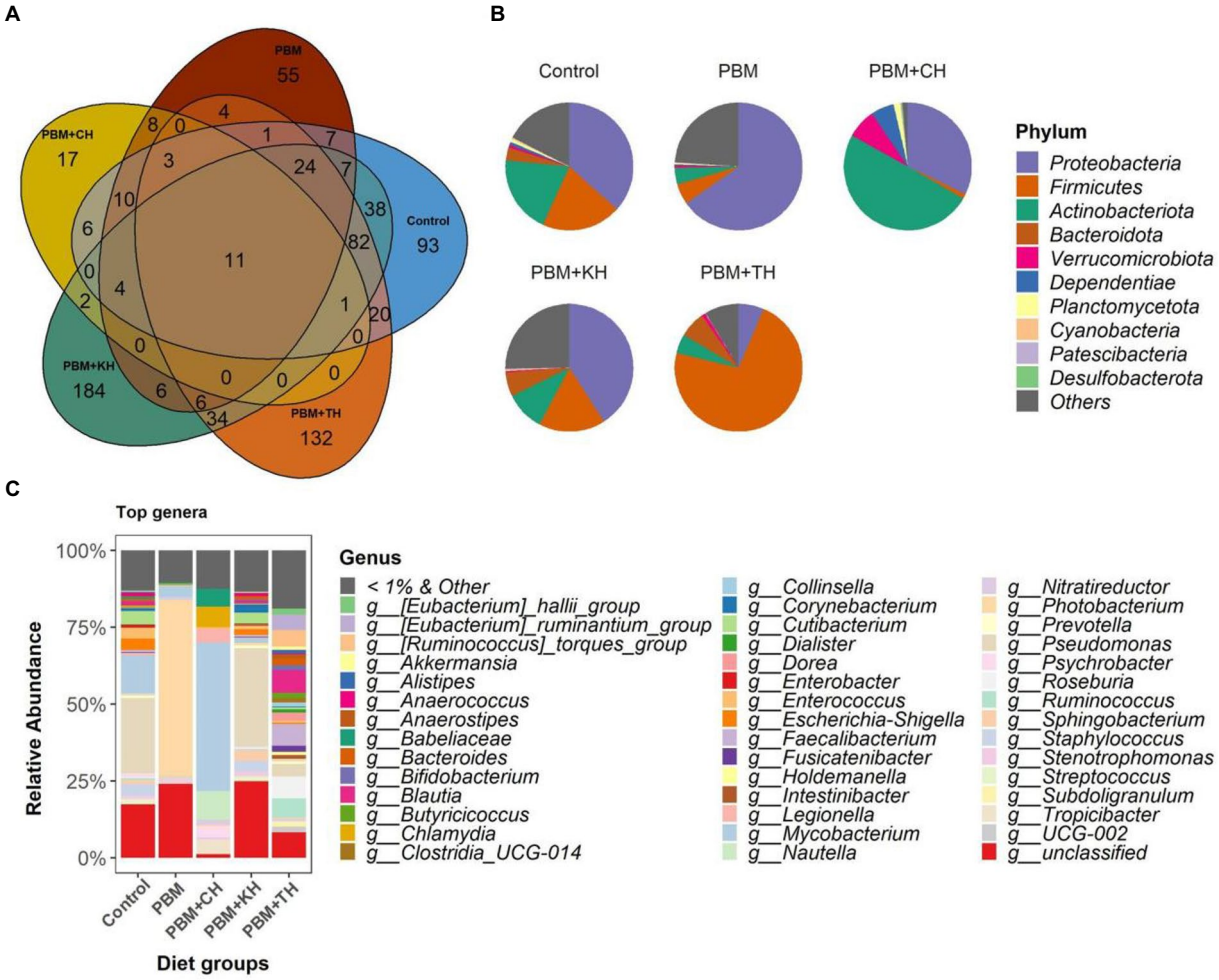
Gut bacterial composition in the intestine of barramundi fed FM-based control, PBM alone or supplemented with various FPHs supplemented diet over a period of 42 days. **(A)** The number of shared, unique and core genera in five different feeding groups. **(B)** Pie-chart representing the relative abundance of bacteria at phylum-level. **(C)** Relative abundance of top 1% genera in five different feeding groups.

From the 146 identified genera, 106 were found to be unique in the PHM + TH and PBM + CH diet groups suggesting the colonization of new bacteria with these two diets. Among the classified ASVs, *Strenotrophomonas* was found distributed in more than 90% of samples irrespective of diet groups; and, therefore, can be defined as core genera of juvenile barramundi (Figure 4A). Proteobacteria was found dominant in control, PBM and PBM + KH diets, wherein more than 60% of reads were classified to Proteobacteria in the fish gut from the PBM diet. Fish gut had Actinobacteria-rich communities with the PBM + CH diet, whereas 72% of reads were assigned to *Firmicutes* for the PBM + TH diet group (Figure 4B), higher *Pseudomonas* abundance was detected at the genus level in the control (24.2%) and PBM + KH (34.6%) groups. *Photobacterium* (54.6%) and *Mycobacterium* (42.4%) were dominant in the PBM and PBM + CH groups, respectively (Figure 4C). No single dominancy was observed in the PBM + TH group with the *Ruminococcus* group represented 11% of the sequences.

**Figure 5:**
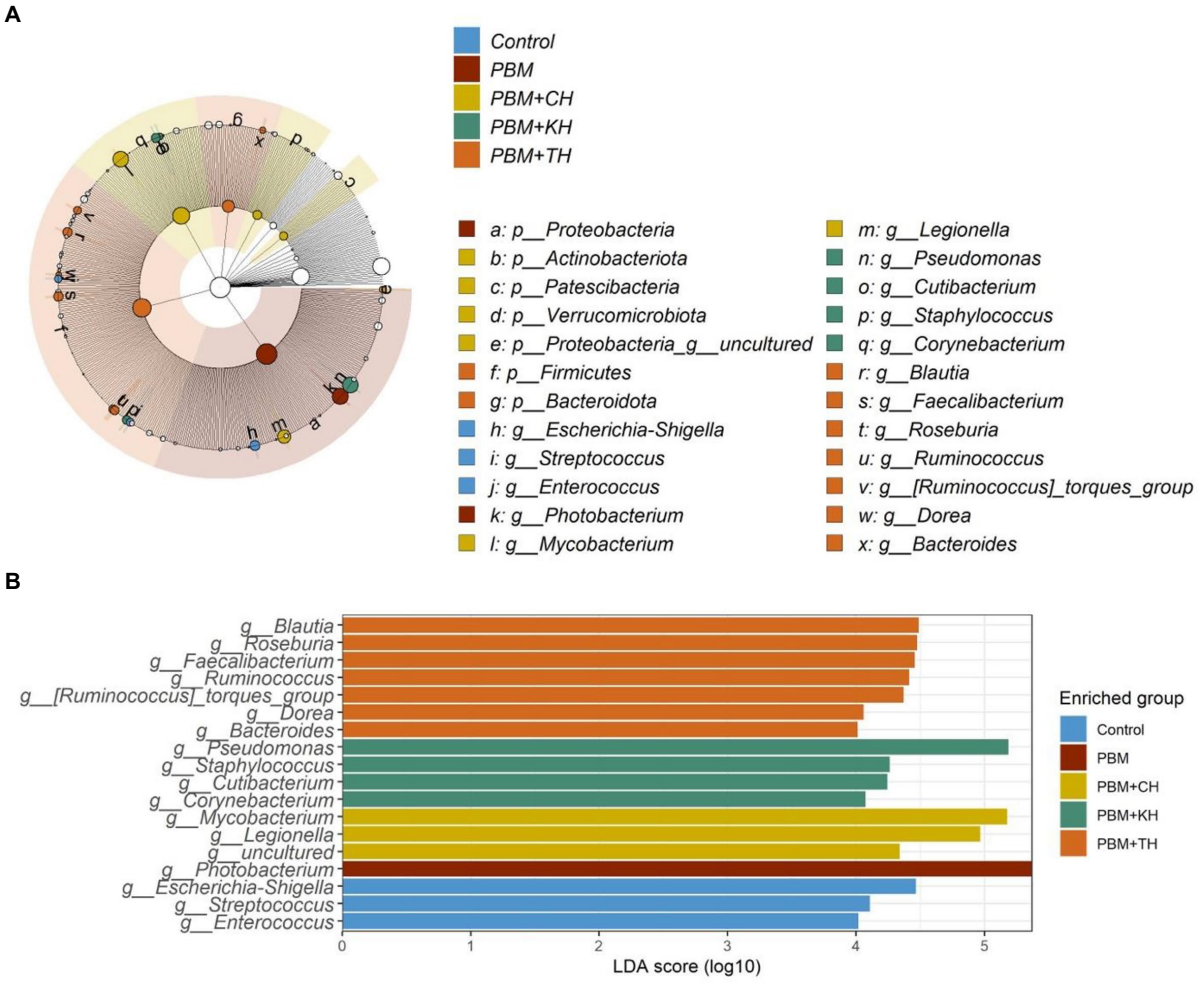
Differential abundance of bacterial communities in barramundi fed FM-based control, PBM alone or supplemented with various FPHs supplemented diet over a period of 42 days. LDA value of ≥4.0 and *p value* > 0.05 were considered for differential abundance analysis.

**Figure 6:**
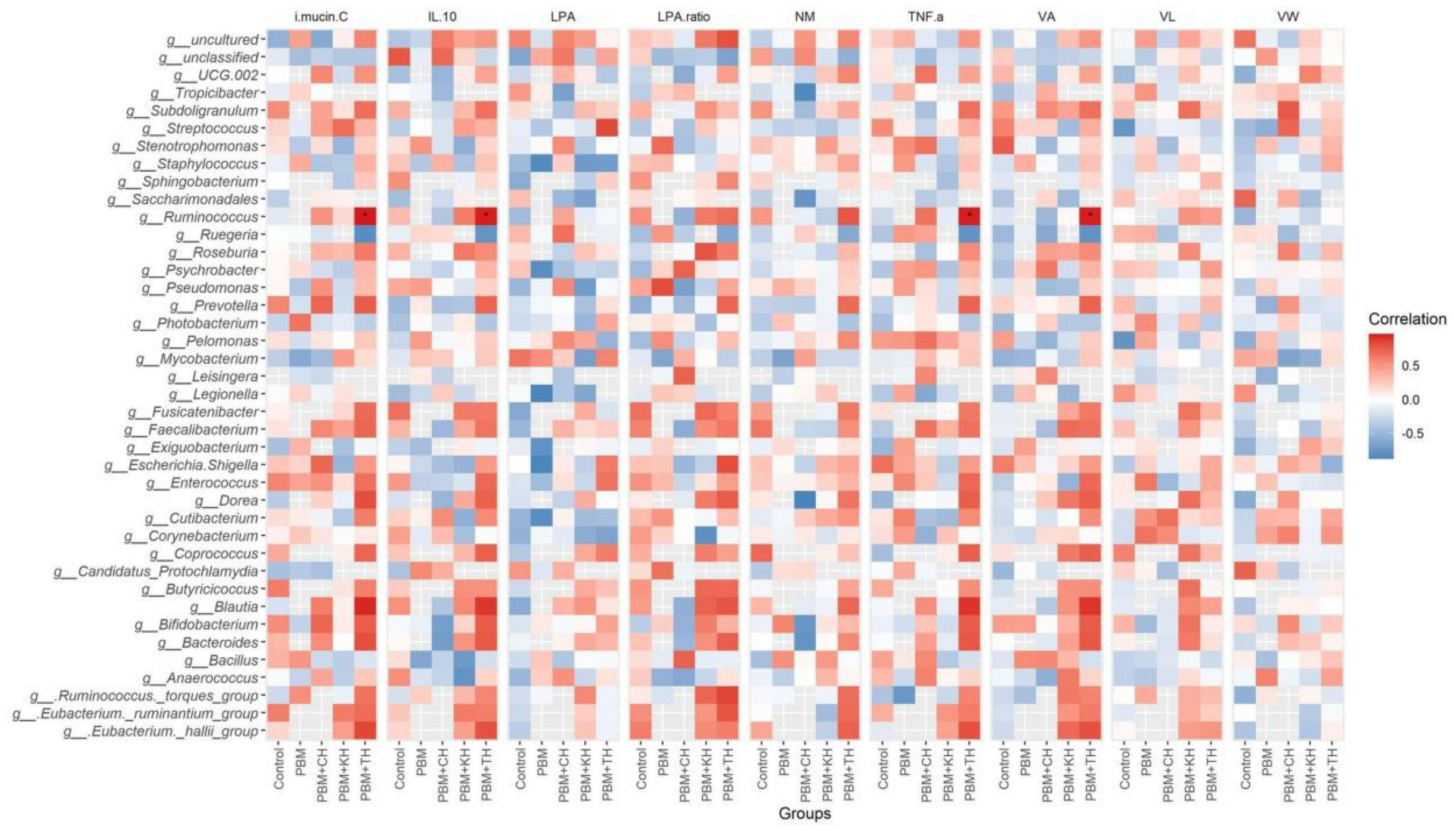
Pearson correlation plot of taxa-fish health indices. The top 40 bacterial genera were considered for correlation analysis with end-trial metadata for fish health and immunity. Red represents a positive correlation, whereas blue indicates a negative correlation. ^*^Significant correlation at α-level of 0.05.

Linear Discriminant Analysis Effect Sizes-based differential abundance identified 24 taxa at phylum and genus levels with significantly higher abundance in five different feeding groups. Higher Proteobacterial abundance in the PBM diet and *Firmicutes* abundance in the PBM + TH group was detected. At the genus level, the control group had a significantly higher abundance of *Escherichia-Shigella*, *Streptococcus*, and *Enterococcus*. Significantly higher *Photobacterium* with PBM, *Staphylococcus* and *Pseudomonas* with PBM + KH and *Mycobacterium* and *Legionella* with PBM + CH diets were observed when compared to the other treatments. Finally, the PBM + TH diet augmented the abundance of *Ruminococcus*, *Faecalibacterium*, and *Bacteroides* in the aligned fish gut (Figures 5A,B). *Ruminococcus* was positively associated with the *i mucin c* and inflammatory cytokines (*tnf-α and il-10*) in the intestine of the PBM-TH fed barramundi group (*p* < 0.05), while other mucosal barrier functions (LPA, LPA raito, NM, VL, and VW) showed no correlation with any genus in barramundi fed all test diets (*p* > 0.05), as determined by Pearson correlation (Figure 6).

### Muscle health

**Figure 7:**
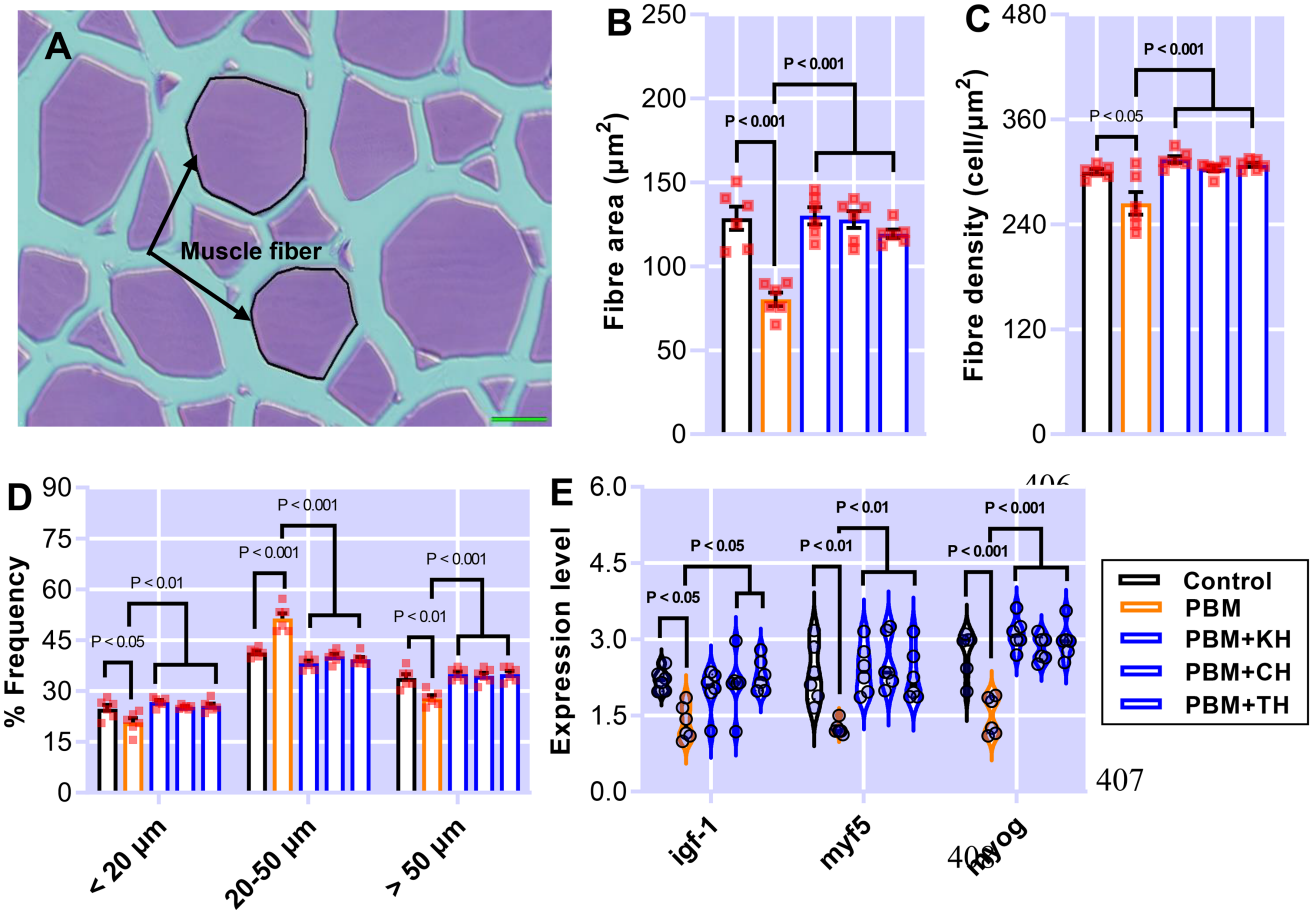
The representative histological micrograph of muscle showing the measurements of muscle fiber **(A)**; the comparison of quantitative measurements of muscle fiber **(B,C)** and the frequency percentage of hyperplasia and hypertrophy **(D)**; and fold change in the expression of muscle growth relevant genes **(E)** of barramundi fed FM-based control, PBM alone or supplemented with various FPHs supplemented diet over a period of 42 days.

Muscle measurement and quantification, and muscle-health-relevant gene expression, are presented in Figures 7A–E. Muscle fiber area (Figure 7B; *p* < 0.001) and density (Figure 7C; *p* < 0.05) decreased significantly in barramundi-fed PBM-based diets, but supplementation of various FPHs improved fiber area and density when compared with the PBM-fed group (Figures 7B,C; *p* < 0.001). PBM-fed barramundi showed a lower number of <20 μm muscle fibers (hyperplasia; *p* < 0.05) which improved in barramundi when fish were fed PBM supplemented with FPHs (Figure 7D; *p* < 0.01). However, when compared with the control diet, 20–50 μm muscle fibers were enhanced in barramundi-fed PBM (*p* < 0.001). 20–50 μm muscle fibers decreased in barramundi fed FPHs supplemented PBM diet when compared to those fed the PBM-based diet (*p* < 0.001) but were comparable to the control-fed barramundi (*p* > 0.05). Similar to hyperplasia, hypertrophy (>50 μm) was significantly impacted by PBM (*p* < 0.01); however, the negative effect of PBM was attenuated when barramundi was fed FPHs-supplemented PBM diets (*p* < 0.001). There was no difference in the hypertrophy measurements of barramundi-fed control and FPHs-supplemented diets (*p* > 0.05). Though the expression of *igf-1 I* (*p* < 0.05), *myf5* (*p* < 0.01), and *myog* (*p* < 0.001) were negatively impacted by the PBM based diet, supplementation of various FPHs prevented the PBM-induced negative effects on the expression levels of these genes (*p* < 0.05, 0.01, and 0.001). The expression of *igf-1*, *myf5* and *myog* did not differ between the control and the FPHs-supplemented PBM fed barramundi (*p* > 0.05).

### Serum metabolites

**Figure 8:**
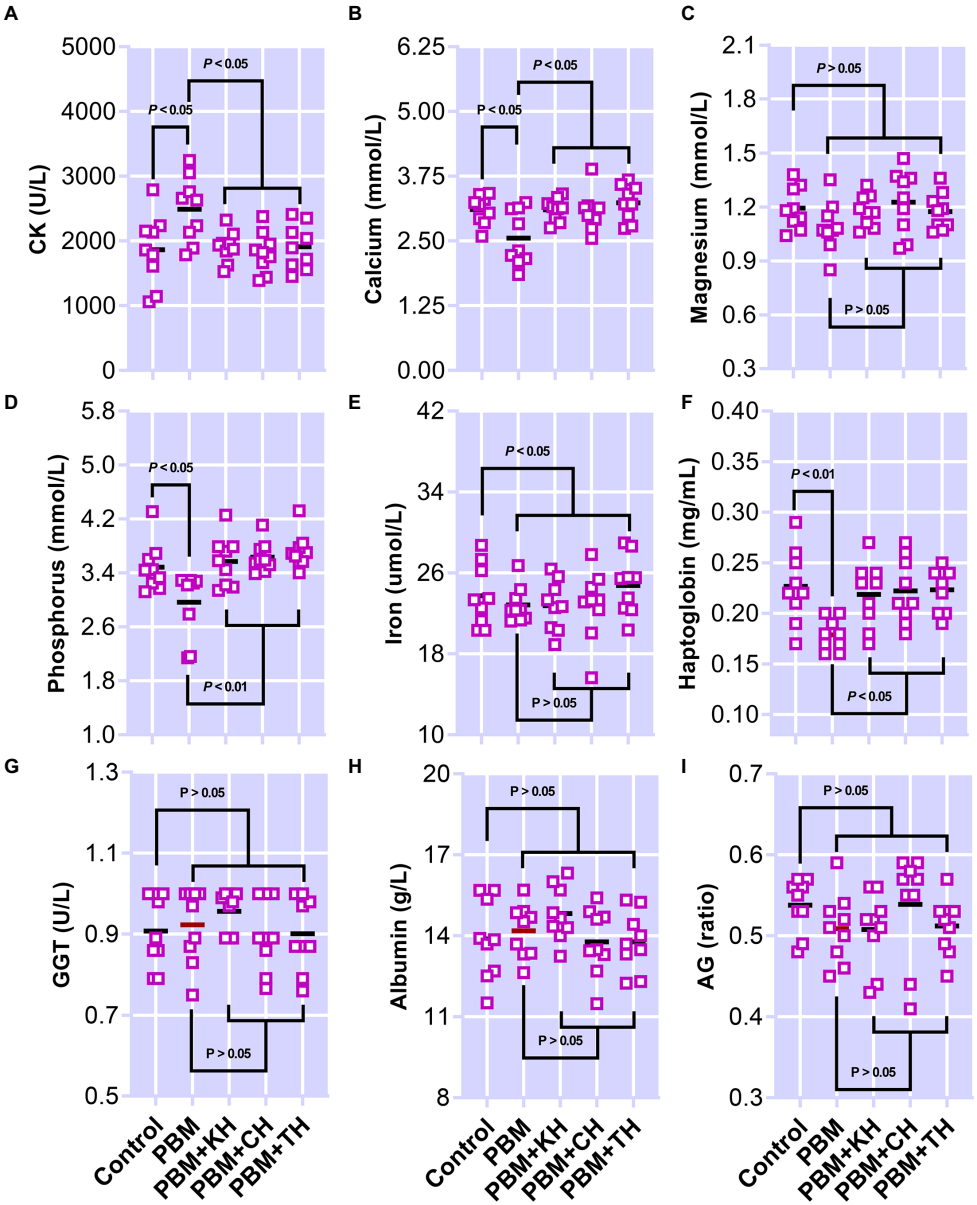
Serum Creatine kinase (CK; **A**), calcium **(B)**, Magnesium (Mg; **C**), inorganic phosphate **(D)**, iron **(E)**, haptoglobin **(F)**, gamma-glutamyltransferase (GGT; **G**), albumin **(H)**, and AG **(I)** of barramundi fed FM-based control, PBM alone, or supplemented with various FPHs supplemented diet over a period of 42 days.

The effect of PBM and PBM-FPHs diets on a panel of serum metabolites is presented in Figure 8. Creatine kinase (CK; Figure 8A) elevated significantly in fish fed the PBM diet when compared to the control (*p* < 0.05), but there was no variation between control and PBM-FPHs fed barramundi (*p* > 0.05). However, compared to those fed only PBM, FPHs supplementation with PBM attenuated CK levels in barramundi (*p* < 0.05). PBM negatively impacted the levels of calcium and phosphorous (*p* < 0.05) in barramundi serum which was restored when PBM was supplemented with FPHs (*p* < 0.05 and 0.01). Barramundi-fed PBM-FPHs showed no variation in the levels of calcium and magnesium when compared with the control (*p* > 0.05). Haptoglobin levels demonstrated a similar trend (*p* < 0.05 and 0.01). Meanwhile, the different experimental diets did not affect on phosphate, iron, GGT, albumin, and AG ratio (*p* > 0.05).

## Discussion

Supplementing immunostimulants, such as FPHs, in the aquafeed formulation is being used to ameliorate the impact of sub-optimal alternative protein sources, including the prevention of intestinal dysbiosis (1, 7). Replacing FM protein with PBM damaged the general architecture of the barramundi intestine, as proven by the reported atrophy of mucosal fold and reduction of microvilli height and width (11). However, the effects of PBM alone or in combination with FPHs on a wide range of intestinal mucosal barriers in relation to intestinal microbiota and cytokine expression have not been previously reported and thoroughly investigated.

One of the main consequences of replacing high levels of FM with alternative protein sources is gut enteritis resulting from the widening of the lamina propria area (LPA) and shortening of the mucosal folds, subsequently to be associated with intestinal inflammation (37, 38). Such consequences have previously been reported in the gut of barramundi and other carnivorous fish species fed alternative proteins such as PBM and soybean meal (11, 39). In the present study, such an elevation in LPA, and consequently enteritis, in the intestine was found in barramundi-fed a PBM-based diet. The damaging effect of PBM-only diets on intestinal structure were further validated by the lower expression of anti-inflammatory cytokines and the presence of low diversity of microbiota. These negative effects were countered when PBM was supplemented with various FPHs. A similar LPA and LPA ratio in the barramundi fed FPHs supplemented PBM diets to the positive control diets was observed, suggesting that FPHs supplementation could prevent or decline the occurrence of intestinal enteritis. Apart from minimizing intestinal enteritis, FPHs supplementation with PBM increased the villus length and width associated with the absorptive surface area. This may explain the comparable growth performance of barramundi-fed FPHs supplemented diets when compared to those fed a control FM-based diet in an earlier study (1). Similarly, dietary inclusion of tilapia and shrimp hydrolysates separately or concurrently prevented intestinal dysbiosis induced by a low FM-diet and intestinal mucosal health was even better than fish fed a high FM-based diet (40).

The mechanism for enhanced performance when FPHs are included is likely due to various reasons and is therefore difficult to explain. FPHs contain peptides with a shortened length and free amino acids that have been reported to enhance palatability and nutrient absorption by stimulating protein synthesis *via* the activation of the TOR pathway and inhibition of the AAR pathway. These pathways regulation were previously reported to facilitate gastrointestinal development by improving microbiota and regulating inflammatory cytokines expression in largemouth bass, *Micropterus salmoides* (41).

A good number of studies have found a potential anti-inflammatory effect of FPH in both *in vivo* and *in vitro* conditions, coupled with the reduction of the expression levels of pro-inflammatory cytokines, such as *tnf-α*, *il-6*, *il-1β*, and *il-8* (42–44). Intestinal inflammation has been reported to be associated with the higher expression of pro-inflammatory, with a concurrent lower expression of anti-inflammatory cytokines in fish (45). For example, replacing FM with 50 and 75% of soybean meal stimulated gut inflammation by elevating the expression of pro-inflammatory cytokines (*tnf-α*, *il-1β*, *il-2*, and *il-8*) and lowering the expression of anti-inflammatory gene *il-4* (46). A similar result was found in the present study, where the PBM-based diet enhanced the expression of *tnf-α* and reduced the expression of *il-10*, aligning with the results of the LPA and LPA ratio, thereby aggravating intestinal inflammation. A contrasting and balanced inflammatory response was found in the intestine of barramundi when the PBM was supplemented with various FPHs. This was supported by a higher expression of both *tnf-α* and *il-10* in PBM-FPH fed groups when compared to those fed PBM-based diet, and the results were comparable to fish fed the FM control diet. Dietary supplementation of FPHs provided a similar beneficial effect on inflammatory responses in the intestine of larval largemouth bass, manifested by the downregulation of pro-inflammatory cytokines (*5-lox* and *il-8*) and upregulation of anti-inflammatory cytokines (*il-10*) (22). These authors reported that the presence of high amounts of free histidine and glycine in FPHs supressed the inflammatory response in largemouth bass. Also, the inhibitory effects of anti-inflammatory cytokines on the expression of pro-inflammatory cytokines have been reported to be suppressed in the inflammatory response in some teleost fish (22).

Poultry by-product meal supplemented with black soldier fly larvae meal, and FPHs were reported to influence the mucins production, in particular, acidic and neutral mucin, in the gut of barramundi (1, 8). The association between mucin and mucin-relevant production genes, such as *i-mucin c*, and microbial genera composition has been reported for the first in the present study. Similar to neutral mucin production in the intestine of barramundi, the expression of *i-mucin c* was aggravated by the PBM-based diet but restored in the intestine of barramundi-fed PBM-FPH diets, suggesting that supplementation of FPHs could prevent PBM-induced gut mucosal dysfunction thereby maintaining gut homeostasis.

Gut microbial diversity has also been reported to be associated with gut homeostasis (47). Alternative protein ingredients in aquafeed are reported to often reduce bacterial diversity and thus compromise the health of farmed fishes (25). Although the evaluation of gut microbial composition in response to alternative finfish aquafeed protein sources has been gaining importance in the evaluation of gut health of farmed fish, understanding the interaction between gut microbial composition and mucosal epithelial barriers and associated genes has largely been overlooked. In this study, gut microbial diversity were negatively impacted by the PBM-based diet along with an enrichment of the *Photobacterium* genus. The prevalence of this genus was recently reported to increase in the gut system of barramundi when exposed to a variety of environmental stresses (48). In the present study, the observed bacterial dysbiosis in the intestine of barramundi fed PBM protein only was aligned with the results of the negatively altered mucosal barriers and expression of inflammatory cytokines and *i-mucin c*. Thus, the PBM-only based diet compromised gut homeostasis. It has previously been reported that rich bacterial diversity has been associated with a number of beneficial roles in fish health: stimulation of innate immunity, out-competition of pathogens for nutrients and colonization and production of antimicrobial compounds (e.g., bacteriocins, peptides and proteins). These functions deprive pathogenic bacteria in the gut surface for the establishment, resulting in resistance to pathogen invasion and intestinal infection (8, 49–51).

Fish protein hydrolysates supplementation with PBM restored diverse beneficial microbial composition, as proven by a significantly higher microbial diversity in barramundi-fed PBM-FPHs diets when compared to those fed PBM-based diet. The reasons for this improvement may be multiple. For example, peptides from the hydrolysis process contain a rich source of essential amino acids (not analyzed in the current study), which have been reported to act as natural sources of nitrogen essential for microbiota growth (27). Enzymatic hydrolysis in the present study also produced more than 90% of smaller peptides, which may also be related to the beneficial effect of FPHs on gut microbiota. Notably, a high gut microbial diversity with increased prevalence of *Ruminococcus*, *Faecalibacterium*, and *Bacteroides* in barramundi fed the PBM-TH diet suggested further beneficial effects of TH supplementation specifically. A substantial enrichment in the composition of *Ruminococcus* in mice fed *Spirulina platensis* protease hydrolysate for 8 weeks has also been reported (52). Our previous studies found an enrichment of *Ruminococcus* in the gut of barramundi-fed PBM diets supplemented with black soldier fly larvae meal alone or in combination with TH (3, 8). *Ruminococcus* produces short-chain fatty acids which play an important role in the degradation of indigestible carbohydrates, such as resistant starch (53) and dietary fibers, and have been reported to mitigate metabolic disorders caused by high-lipid diets (54–56). Also, *Ruminococcus* species were used as a probiotic (Zado®) and reportedly boosted the overall health of Nile tilapia, *Oreochromis niloticus* (57). This probiotic effect may explain the strong correlation between the *Ruminococcus* prevalence and mucosal barrier functions and inflammatory cytokines in the present study. Similarly, *Faecalibacterium* is the most important commensal butyrate-producing bacterium, which was abundantly found in healthy shrimp (58) and is considered a bio-indicator of human health (59). *Bacteroides strain* has been documented as a healthy bacteria in grass carp, *Ctenopharyngodon idella* and also has been reported to alleviate lipopolysaccharide-induced inflammation in mice (60). Thus, these genera may contribute to the more efficient energy utilization of feed, and improved intestinal health of the host by producing butyrate as an end product of dietary fiber fermentation and by promoting mucosal barrier functions. The beneficial effect of dietary FPHs on proliferating the abundance of beneficial bacteria (*Plesiomonas*) and reducing the prevalence of harmful bacteria (*Staphylococcus*) was previously observed in the intestine of larval largemouth bass, *Micropterus salmoides* (22). This was concomitant with the observant mucosal barrier results (VA, VL, VW, and NM).

The gut inflammatory response could be associated with the gut microbiota, promoting the health of farmed fish. For instance, the expression of *il-10* in the anterior intestine was positively associated with the abundance *Serratia nematodiphila*, while *tnf-α* expression was positively associated with the abundance of *Paracoccus yeei in* Gilthead Sea Bream, *Sparus aurata* juveniles fed processed and non-processed animal protein (61). A similar positive association between *Ruminococcus* and *tnf-α* and *il-10* was found in barramundi-fed PBM-TH in the present study. Also, in our study, a positive association of *Ruminococcus* with the expression of i-mucin c in the gut of barramundi-fed PBM-TH was observed, suggesting that the abundance of the beneficial *Ruminococcus*, further increased by TH supplementation, played a role in maintaining the gut homeostasis by balancing inflammatory responses and enhancing mucus production. A higher proportion of small peptides in TH than the other FPH’s might have provided more beneficial effects on the barramundi gut.

Fish growth is directly linked to muscle growth which results from the hyperplasia and hypertrophy of muscle fibers (33, 62). Quantification of histological muscle fiber morphology is one of the important determinants to understand fish muscle growth in aquafeed nutrition research. This quantification provides information on the recruitment of new muscle fibers (hyperplasia, < 20 μm) and hypertrophy (> 50 μm) of existing muscle which has been reported to be influenced by dietary modifications (33). The effect of FM-free diets on muscle fiber morphology aligned with the expression of muscle growth-regulating genes has been evaluated for the first time in this study. The present study found a decline in the size and number of muscle fibers and frequency of hyperplasia and hypertrophy in barramundi fed the PBM-based diet, suggesting that complete replacement of FM with PBM negatively impacted muscle growth. This negative impact can be alleviated in barramundi when various FPHs were supplemented with PBM, which was validated by a significant increase in the muscle fiber frequency in barramundi-fed FPH supplemented PBM, which was comparable to the control. Similarly, the incorporation of 12% of FPH improved muscle growth by regulating muscle microstructure and ultrastructure and muscle growth-related gene expression in turbot (63).

Insulin-like growth factor (*igf*) genes are growth hormones encoding the highly conserved growth regulatory peptide *igf-1*. *igf-1* is used as a biomarker in aquaculture research and is correlated with classical parameters such as growth to understand better the nutritional status of experimental fish (64). The quality of dietary protein has been reported to affect *igf-1* expression by functioning as a mediator in muscle to promote growth and inhibit protein degradation, thereby affecting the growth of fish (65). The lower expression of *igf-1* in barramundi-fed PBM in the present study, aligning with poor growth performance in our previous study (11), suggested that a PBM-based diet was not palatable, resulting in starvation which might have loosened the sensitivity of muscle tissues to produce *igf*. This was further validated by lower feed intake in barramundi fed the PBM-based diet (11). Similar to *igf*, fish growth is also regulated by myogenic regulatory factors such as (MRF) and myostatin (MSTN), directly stimulating muscle proliferation, differentiation and hypertrophy (66). myf5 and *myog* are specific MRF that regulate myogenesis by contributing to muscle cell proliferation and initiation and maintenance of the differentiation programs (67). As reported here the PBM-based diet down-regulated the expression levels of *myf5* and *myog*, suggesting that PBM could negatively impact the hyperplasia and hypertrophy of barramundi muscle by regulating the expression of *igf* and MRF.

Supplementation of 10% of various FPHs alleviated the negative effect of the PBM-only diet on the muscle growth of barramundi. This was supported by significantly higher expression of *igf* and MRF in the muscle of barramundi-fed PBM diets supplemented with FPHs when compared to those fed the PBM-based diet. The expression levels of *igf* and MRF in barramundi fed FPHs supplemented diets were similar to those fed the FM-based control diet. Such attenuation of PBM-induced negative impact on muscle health by FPHs supplementation could be due to the presence of functional molecules or aligned with the amino acids content in FPHs. Such functional molecules in FPHs have been related to increasing plasma *igf-i* levels and liver *igf-i* mRNA expression of fish (68). Two recent studies found that supplementing marine protein hydrolysates and hydrolysed fish protein powder elevated the expression levels of *igf-1* aligned with an increased upregulation of TOR pathway-related genes (41, 69). Hence FPHs could be used to prevent the negative effects of FM omission and alternative protein source replacement in barramundi diets.

Investigation of blood biochemistry parameters has been a powerful analytical tool commonly used in aquaculture studies to monitor the health status of experimental aquacultured animals. Muscle health has been reported to be associated with the levels of CK in barramundi blood, which have been reported to be elevated in barramundi when fed alternative proteins such as soybean meal and lupin meal (31, 32). In this study, a PBM-based diet increased the CK level, aligning with aggravated muscle growth-relevant gene expression. Also, our previous study found histopathological changes manifested by myotome necrosis in the muscle of barramundi fed a PBM-based diet (11). However, CK activity appeared normal when the PBM diet was supplemented with FPHs, suggesting the alleviation of PBM-induced negative effects on barramundi muscle health. Some minerals are good indicators of the secondary phase of stress response in fish, as levels can be decreased by stress such as starvation and malnutrition (70–72), and this can indicate pathological situations (73) in fish blood. A significant drop in the levels of calcium and total phosphorus in the serum of barramundi fed the PBM diet when compared to the control diet suggested that barramundi fed PBM might have suffered from malnutrition or starvation. This was supported by a lower food intake and various pathological indicators (e.g., hepatic steatosis, gill hyperplasia and muscle necrosis) as previously reported (11). An elevation of these minerals in the PBM-FPH-fed barramundi to levels comparable to the control, when compared to the PBM-only treatment, indicated the alleviating capacity of FPHs to prevent the PBM-induced negative effects on mineral levels and content in barramundi serum. Magnesium levels were not altered by the test diets, suggesting that barramundi might have uptaken magnesium from the water, as previously reported in many marine species (70, 74).

Haptoglobin (Hp), a protein that binds with free hemoglobin, has been reported to be associated with lipids and protein oxidation thereby causing damage in surrounding tissue (75). The formation of a complex between haptoglobin and hemoglobin inhibits free hemoglobin-mediated tissue damage (76). For instance, renal failure was found in mice lacking haptoglobin (77). In the present study, barramundi fed the PBM diet, which lacks haptoglobin, when compared to fish fed the FM control diet, might have suggested that PBM inclusion stimulated oxidative damage in barramundi. This notion was supported by a negative effect of the PBM diet on GPx and MDA activity (11). However, FPHs supplementation with PBM restored the levels of Hp, underpinning the antioxidant activity of FPHs, thereby repairing the tissue damage. The remaining serum metabolites which were analyzed remained unchanged and within the normal range of barramundi health status.

In summary, the PBM-only based diet aggravated intestinal homeostasis by impacting intestinal mucosal barrier function, inflammatory response, and microbial composition. Supplementation of 10% of various FPHs, including KH, CH, and TH, obtained from enzymatic hydrolysis of fish waste, could prevent PBM-induced gut enteritis, manifested by quantifying LP and LPA ratio and inflammation in the intestine of barramundi. Also, various FPHs supplementation with PBM restored gut microbial composition and balanced the inflammatory response. In particular, TH supplementation improved further the intestinal mucosal barrier functions (VA, VL, VW, and NM), supported by a significant improvement in microbial diversity with significant enrichment in the *Firmicutes*, *Ruminococcus*, *Faecalibacterium*, and *Bacteroides* genera. The abundance of *Ruminococcus* may have positively influenced the inflammatory cytokines, mucin production relevant genes and VA in the barramundi intestine. Muscle atrophy was triggered by the PBM-based diet, but this effect was improved with FPHs supplementation, as demonstrated by a significant increase in muscle fiber quantification (fiber density, hyperplasia, and hypertrophy) and muscle health relevant gene expression (IGF-1, myf5, and MyoG). Hence, FPHs could be used as supplementary functional ingredients with low-quality alternative protein sources to prevent PBM-induced negative effects on intestinal homeostasis and muscle health, as well as providing a good option in increasing circularity and resource utilization in the aquaculture industry. However, further commercially relevant long-term studies are needed to confirm the beneficial supplementation effect of FPH with PBM in the barramundi diet along with final product quality to ensure product quality produced on raw material sourced from the circular economy is the same.

## Data availability statement

The microbiome datasets presented in this study can be found in online repositories. The names of the repository/repositories and accession number(s) can be found at: https://www.ncbi.nlm.nih.gov/, PRJNA909982.

## Ethics statement

The animal study was reviewed and approved by Curtin University Animal Ethics Committee.

## Author contributions

MC: conceptualization, methodology, formal analysis, investigation, data curation, writing—original draft, and visualization. JH: supervision, writing—review and editing, and project administration. MF, MH, and HA-L: formal analysis and writing—review and editing. RF: supervision, conceptualization, and writing—review and editing. All authors contributed to the article and approved the submitted version.

## Funding

This work was supported by a Research Training Program (RTP) stipend scholarship, funded by the Australian Government, to MC (No. 19061054-Curtin).

## Conflict of interest

The authors declare that the research was conducted in the absence of any commercial or financial relationships that could be construed as a potential conflict of interest.

## Publisher’s note

All claims expressed in this article are solely those of the authors and do not necessarily represent those of their affiliated organizations, or those of the publisher, the editors and the reviewers. Any product that may be evaluated in this article, or claim that may be made by its manufacturer, is not guaranteed or endorsed by the publisher.

## Footnotes

^1^ https://github.com/umerijaz/microbiomeSeq

^2^ https://github.com/microbiome/microbiome
